# Predicting the 90-day prognosis of stereotactic brain hemorrhage patients by multiple machine learning using radiomic features combined with clinical features

**DOI:** 10.3389/fsurg.2024.1344263

**Published:** 2024-02-08

**Authors:** Jinwei Li, Cong Liang, Junsun Dang, Yang Zhang, Hongmou Chen, Xianlei Yan, Quan Liu

**Affiliations:** ^1^ Department of Neurosurgery, Liuzhou Workers Hospital, Liuzhou, Guangxi, China; ^2^ Department of Neurosurgery, West China Hospital, Sichuan University, Chengdu, Sichuan, China; ^3^ Department of Pharmacy, Liuzhou Workers Hospital, Liuzhou, Guangxi, China; ^4^ Department of Vascular Surgery, Fuwai Yunnan Cardiovascular Hospital, Affiliated Cardiovascular Hospital of Kunming Medical University, Kunming, Yunnan, China

**Keywords:** hypertensive cerebral hemorrhage, radiomic features, predictive model, machine learning, stereotactic hematoma removal

## Abstract

Hypertensive Intracerebral Hemorrhage (HICH) is one of the most common types of cerebral hemorrhage with a high mortality and disability rate. Currently, preoperative non-contrast computed tomography (NCCT) scanning-guided stereotactic hematoma removal has achieved good results in treating HICH, but some patients still have poor prognoses. This study collected relevant clinical and radiomic data by retrospectively collecting and analyzing 432 patients who underwent stereotactic hematoma removal for HICH from January 2017 to December 2020 at the Liuzhou Workers Hospital. The prognosis of patients after 90 days was judged by the modified Rankin Scale (mRS) scale and divided into the good prognosis group (mRS ≤ 3) and the poor prognosis group (mRS > 3). The 268 patients were randomly divided into training and test sets in the ratio of 8:2, with 214 patients in the training set and 54 patients in the test set. The least absolute shrinkage and selection operator (Lasso) was used to screen radiomics features. They were combining clinical features and radiomic features to build a joint prediction model of the nomogram. The AUCs of the clinical model for predicting different prognoses of patients undergoing stereotactic HICH were 0.957 and 0.922 in the training and test sets, respectively, while the AUCs of the radiomics model were 0.932 and 0.770, respectively, and the AUCs of the combined prediction model for building a nomogram were 0.987 and 0.932, respectively. Compared with a single clinical or radiological model, the nomogram constructed by fusing clinical variables and radiomic features could better identify the prognosis of HICH patients undergoing stereotactic hematoma removal after 90 days.

## 1 Introduction

Hypertensive intracerebral hemorrhage (HICH) is a type of spontaneous non-traumatic cerebral hemorrhagic disease and is the most common cerebrovascular disease in middle-aged and elderly people. It is caused by rupture and bleeding of small cerebral arteries caused by long-term cerebral atherosclerosis and hypertensive lesions (1, 2). After the onset of the disease, patients usually present with limb dysfunction, hemiparesis, and even death in severe cases. Theoretically, early surgery is a reasonable treatment method to both directly address the occupying effect of the hematoma and to remove the hematoma and the various chemical damage factors released by the metabolism and action of the hematoma (3). With the continuous development of minimally invasive technology, timely acceptance of minimally invasive intracranial hematoma removal can effectively control the further development of the disease and reduce the mortality and disability rate to a certain extent (4). Therefore, minimally invasive surgical treatment in early HICH patients may help the prognosis of neurological function recovery.

With the continuous progress of clinical treatment, more and more ways of hematoma removal have been developed. Based on traditional craniotomy hematoma removal, minimally invasive craniotomy with small windows, stereotactic hematoma removal, endoscopic hematoma removal, robot-assisted hematoma removal, and other treatment methods have been carried out successively (5–8). Computed Tomography (CT) guided stereotactic hematoma removal was one of the main protocols for clinical treatment of hypertensive cerebral hemorrhage, and has partially replaced craniotomy for patients with cerebral hemorrhage with a hematoma volume of 20–40 ml, accompanied by neurological deficits and a mild degree of impaired consciousness (9). It also had the advantages of less surgical trauma, more complete hematoma removal, and faster postoperative recovery of patients, and is now widely used in clinical practice (9–11). Previous studies have found that early performance of stereotactic hematoma removal is more beneficial for patients’ prognosis and recovery (12). Although the majority of patients undergoing stereotactic hematoma removal have achieved good outcomes, a proportion of patients with HICH still have a poor prognosis or even die after comprehensive treatment. Therefore, there is an urgent need to investigate the factors associated with poor prognosis in patients with HICH undergoing stereotactic hematoma removal, which is of great clinical value to improve the prognostic function of patients.

Machine Learning (ML) is increasingly causing a wide range of applications in disease diagnosis and differential diagnosis, efficacy assessment, and prognosis determination due to its wide application and increasing popularity of computational power. ML is an artificial intelligence method for learning patterns and rules from given information (13). Recent studies have applied ML to severity and outcome prediction models for neurological diseases, such as ischemic stroke (13), aneurysmal subarachnoid hemorrhage (14), and traumatic brain injury (15). However, ML approaches in the field of ICH have mainly focused on timely diagnosis and automated volume quantification (16, 17), and algorithms to predict clinical outcomes are lacking. Recently, Wang et al. pioneered the development of ML-based outcome prediction models by combining initial clinical presentation, laboratory data, and imaging presentations, which were limited to brain hemorrhage volume and location, intraventricular hemorrhage, ventricular compression, and midline structural displacement (18). Further integration of disease imaging features may have additional prognostic value (14). Previous studies have demonstrated that specific CT markers and histogram-based analysis of brain hemorrhage heterogeneity are associated with poorer clinical outcomes (19–21). However, most of the current studies have performed radiomic features feature analysis or clinical feature analysis alone to construct machine learning models. Therefore, the modeling of clinical features combined with imaging histological features may be a new modeling approach to help predict the prognosis of patients undergoing stereotactic HICH.

In this study, we hypothesized that clinical features combined with imaging histological features of hematoma may help predict the functional prognosis of patients with stereotactic hypertensive cerebral hemorrhage at 90 days. We compared and analyzed the clinical model constructed by multiple machine learning classifiers, the radiomic features model, and the combined Nomogram model combining the two types of features. To validate and generalize our hypothesis, we develop and validate an NCCT-based clinical-radiomic features nomogram to identify patients with poor prognosis in patients undergoing stereotactic HICH.

## 2 Materials and methods

### 2.1 General clinical information

This study was conducted after review and approval by the Ethics Committee of Liuzhou Workers’ Hospital (KY2023155), and written informed consent from patients has been waived. A total of 432 patients with HICH who underwent CT-guided stereotactic hematoma removal at our hospital from January 2017 to December 2020 were retrospectively collected for clinical and imaging data.

Inclusion criteria: ① Glasgow Coma Scale (GCS) ≥8 on admission, clear history of previous hypertension, and head CT confirmed as patients with cerebral hemorrhage with bleeding sites in the thalamus, internal capsule, lobes, and cerebellum; ② preoperative notification of the condition, different surgical approaches and related risks, and patients voluntarily agreed to undergo stereotactic surgery; ③ age >18 years; ④ preoperative ③ age >18 years old; ④ preoperative imaging and clinical data were not missing.

Exclusion criteria: (i) cerebral hemorrhage caused by cerebral arteriovenous malformation, tumor, or intracranial aneurysm rupture (*n* = 75); (ii) cerebral hemorrhage caused by hematologic disorders and coagulation dysfunction (*n* = 16); (iii) cerebral hemorrhage caused by craniocerebral trauma (*n* = 35); (iv) patients with incomplete clinical and imaging data (*n* = 38). After the above screening and exclusion, a total of 268 patients with HICH who underwent stereotactic hematoma removal were retrospectively analyzed. mRS score scale was used for telephone or outpatient follow-up of patients to assess their functional prognosis 90 days after discharge from the hospital.

The mRS score scale refers to Previous studies of patients with cerebral hemorrhage with an mRS score of 0–3 were considered a good prognosis group and an mRS score of 4–6 was considered a poor prognosis group (Supplementary Table S1). Patients were randomly divided into a training set (*n* = 214) and a test set (*n* = 54) according to an 8:2 ratio. GCS score at admission, age, gender, systolic blood pressure at admission, diastolic blood pressure at admission, blood routine, coagulation function, electrolytes, blood lipids, maximum transverse diameter of the hematoma, volume of the hematoma, site of the hematoma, whether the hematoma was located in the dominant brain, whether it had broken into a ventricle or not, and mRS score at the 90-day post-discharge follow-up were collected from the enrolled patients.

### 2.2 Radiological image acquisition and region of interest outlining

**Figure 1:**
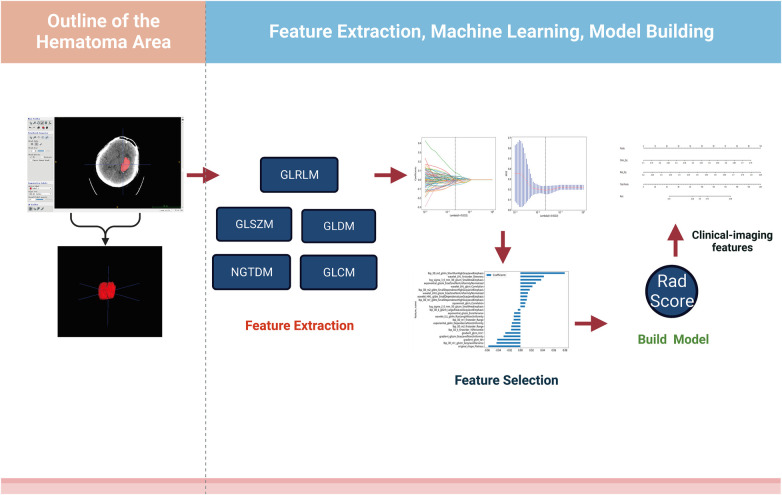
Design roadmap. Steps through feature extraction, machine learning screening, and model construction, respectively.

The analysis flow of this study was shown in Figure 1. All patients were scanned preoperatively using a spiral CT machine (GE Revolution Apex 128-row CT) and a standard scanning protocol (tube voltage 120 kV, tube current 400 mA, matrix size 512 × 512, frame rotation time 0.5 s, field of view 25 cm, layer thickness 5 mm.

**Figure 2:**
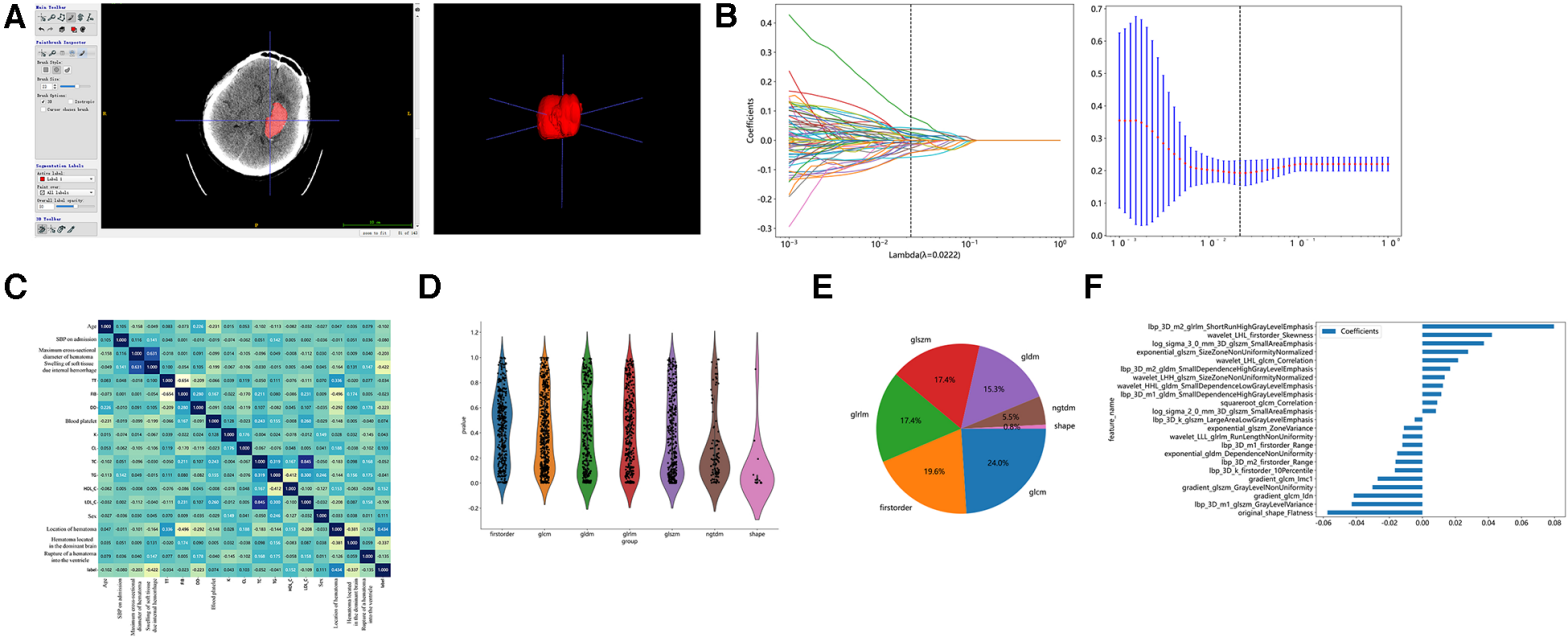
Hematoma region outlining, feature extraction, and screening. (**A**) Outlining interest using the open source tool ITK-SNAP software. (**B**) Imaging histology feature screening using LASSO regression models. (**C**) Comparison of correlations of clinical characteristics. (**D**) Number and proportion of imaging features. (**E**) Screening of Imaging Histological Features. (**E**) Histogram of imaging histology scores based on selected features.

The region of interest (ROI) of the intracranial hematoma was manually outlined on the preoperative CT images using the open source tool ITK-SNAP (version 3.8) software on a level-by-level basis, as follows: (1) Two deputy chief physicians of the Department of Imaging Medicine jointly reviewed the films to determine the boundaries and location of the patient’s hematoma. In case of disagreement, one of the chief radiologists will determine the final boundary: (2) One deputy chief physician (with 10 years of experience) will manually outline the ROI along the edge layer by layer (Figure 2A).

### 2.3 Radiomics feature extraction

The radiomics features can be divided into three categories, including shape-based features, first-order features, and texture features. Shape features describe the three-dimensional shape characteristics of the tumor, such as volume, surface area, and maximum diameter in two and three dimensions. First-order features describe the first-order statistical distribution of voxel intensities within the tumor, which was calculated based on the global gray histogram, including the mean, median, minimum, and maximum values. Texture features describe the second-order and higher-order spatial distributions of voxels. The texture features include gray level cooccurrence matrix (GLCM), gray level run length matrix (GLRLM), gray level size zone matrix (GLSZM), gray level dependence matrix (GLDM), and neighboring gray tone difference matrix (NGTDM), etc. The pyradiomics version 3.0 was used to extract the image histology features. The pyradiomics documentation (https://pyradiomics.readthedocs.io/en/latest) provided the definitions and formulas for all image histology features.

### 2.4 Feature screening process

The Mann–Whitney U statistical test was first performed on all radiomic features, and radiomic features with the *p* value <0.05 were retained. Pairs with Spearman's correlation coefficient greater than 0.9 were identified as highly correlated, and one of the radiomic features with a correlation greater than 0.9 was randomly retained. Finally, the least absolute shrinkage and selection operator (Lasso) model was used on the training set for radiomic features feature screening. A 10-fold cross-validation was used based on the adjustment weight λ, where the final value of λ yielded the minimum (mean square error) MSE value, λ = 0.0222 (Figure 2B).

Subsequently, the radiomics features score for each patient was obtained by a linear combination of the retained features, weighted by their model coefficients, with the following equation (Supplementary Table S2):

$$\begin{array}{c} \mathrm{Rad}\text{-}\mathrm{score}=0.3121951219512192\;++0.030477\;*\;A-0.021783\;\\ {}*\;B\;-\;0.036378\;*\;C\;-0.017005\;*\;D\;-0.031289\;*\;E\;-0.011007\;\\ {}*\;F\;+0.022391\;*\;G\;-0.043688\;*\;H\;-0.031867\;*\;I\;+0.012716\;\\ {}*\;J\;+0.063574\;*\;K\;+0.051280\;*\;L\;-0.042588\;*\;M\;+\\ 0.021230\;*\;n\;+0.006540\;*\;o\;+0.004479\;*\;p\;+0.028441\;\\ {}*\;q\;+0.019110\;*\;r\;-0.002032\;*\;S \end{array}$$

### 2.5 Machine learning algorithms for radiomics model and clinical model construction and validation

The filtered image histology features were input to five ML classifiers for model building, such as Logistic Regression (LR), Support Vector Machine (SVM), k-nearest Neighbor (KNN), Light Gradient Boosting Machine (LightGBM), and Multilayer Perceptron (MLP) were used to build the model. A 5-fold cross-validation was used to optimize the model parameters to reduce model overfitting. Model performance was evaluated using accuracy, sensitivity and specificity, positive prediction rate, negative prediction rate, and receiver operating characteristic curve (ROC) curve and the area under the curve (AUC).

The model construction for clinical features was consistent with the radiomics features modeling process. The included clinical features were analyzed univariately and the clinical variables with statistically significant differences were selected and used to construct the clinical feature models.

Different ML classifiers were compared in the clinical and radiomics models for AUC, respectively. The algorithm LightGBM performed better in the radiomic features and clinical models and did not show the overfitting that often occurs in tree models. Therefore, the radiomic features model and the clinical model using this classifier were compared with the clinical-radiomics model for model performance.

### 2.6 Construction and validation of clinical-radiomics nomograms

To evaluate the prognosis of patients with stereotactic HICH by combining clinical features with radiomics features, a clinical-radiomic nomogram model was constructed. The diagnostic efficacy of the nomogram was also examined in the test set, and ROC curves were plotted to evaluate the diagnostic efficacy of the nomogram. The calibration curves were plotted to evaluate the consistency between the prediction and the actual observation of the prognosis of hypertensive stereotactic patients. Decision Curve Analysis (DCA) was plotted to evaluate the clinical benefit of the prediction model.

### 2.7 Statistical analysis

Statistical analysis was performed using R software (4.1.2). Normally distributed measures were expressed as mean ± standard deviation (x̅ ± s), while differences in the characteristics of clinical variables between the poor prognosis group and the good prognosis group were compared using the independent samples *T*-test or the Mann–Whitney *U* test. Categorical variables were expressed as frequencies or percentages, and differences between the two groups were analyzed using the Chi-square test or Fisher exact test. Differences in AUC between models were tested using the Delong test. The Spearman correlation coefficient was used to calculate the correlation between clinical characteristics. Bilateral *p* < 0.05 was considered statistically.

## 3 Results

### 3.1 Clinical characteristics analysis of stereotactic hypertensive patients

Table 1 showed that a total of 268 patients with stereotactic HICH were included, with 200 males and 68 females. The cases were randomly divided into a training set and a test set in the ratio of 8:2, with 214 cases in the training set (mRS ≤ 3, *n* = 76; mRS > 3, *n* = 138) and 54 cases in the test set (mRS ≤ 3, *n* = 18; mRS > 3, *n* = 36). In the training set, by comparing the factors of clinical variables between the good prognosis group and the poor prognosis group, we found that the preoperative GCS score was (12.89 ± 1.86) in the good prognosis group, and (10.45 ± 2.81) in the poor prognosis group, and the difference was statistically significant (*p* < 0.001). The hematoma volume was (23.09 ± 8.87) ml in the good prognosis group and (32.18 ± 12.56) ml in the poor prognosis group, with a statistically significant difference (*p *< 0.001). The maximum transverse diameter of the hematoma was (5.24 ± 1.01) mm in the good prognosis group and (4.79 ± 1.01) mm in the poor prognosis group, with a statistically significant difference (*p* < 0.001). We also found that whether the hematoma was located in the basal ganglia region and the location of the hematoma in the cerebral hemisphere made a difference in the prognosis of the patients (*p* < 0.001). The remaining clinically relevant information such as age, systolic blood pressure at admission, diastolic blood pressure at admission, coagulation-related tests, glucose, CRP, hemoglobin, platelets, electrolytes, and lipids, and whether the hematoma broke into the ventricle did not show significant differences between the two populations (*p* > 0.05). In the same test set, the same results were obtained, as shown in Table 1. In our study, correlation analysis revealed that whether the hematoma was located in the basal ganglia region, the amount of hematoma, and the location of the hematoma in the cerebral hemisphere correlated with the prognosis of patients with hypertensive cerebral hemorrhage who underwent stereotaxia (*r* > 0.3) (Figure 2C).

**Table 1:** Comparison of baseline information between prognostic and poor prognostic groups in different data sets.

Variables	Training set (*n* = 214)		*P*	Test set (*n* = 54)		*P*
	Poor prognosis group (*n* = 138)	Good prognosis group (*n* = 76)		Poor prognosis group (*n* = 36)	Good prognosis group (*n* = 18)	
Admission GCS score	10.45 ± 2.81	12.89 ± 1.86	**<0**.**001**	10.08 ± 2.97	13.22 ± 1.70	**<0**.**001**
Age (years)	56.72 ± 11.64	53.99 ± 10.91	0.094	58.94 ± 13.44	57.94 ± 14.56	0.803
Gender			0.205			0.078
Female	34 (0.25)	13 (0.17)		17 (0.47)	4 (0.22)	
Male	104 (0.75)	63 (0.83)		19 (0.53)	14 (0.78)	
Systolic blood pressure (mmHg)	176.66 ± 34.17	171.88 ± 27.31	0.296	183.72 ± 33.86	171.67 ± 30.14	0.207
Diastolic blood pressure (mmHg)	107.00 ± 21.25	103.08 ± 18.02	0.175	110.47 ± 23.04	105.72 ± 21.70	0.470
PT (s)	10.65 ± 0.95	10.66 ± 0.80	0.958	10.50 ± 0.71	10.49 ± 0.83	0.959
INR	0.92 ± 0.09	0.92 ± 0.07	0.999	0.91 ± 0.06	0.91 ± 0.07	0.944
APTT (s)	23.01 ± 4.41	22.26 ± 3.89	0.218	22.29 ± 3.62	22.60 ± 2.91	0.757
TT (s)	17.59 ± 1.14	17.45 ± 1.36	0.401	17.99 ± 2.82	18.06 ± 1.47	0.923
FIB (g/L)	3.33 ± 1.31	4.10 ± 6.86	0.200	2.90 ± 0.87	2.79 ± 1.19	0.693
DD (mg/L)	1.87 ± 5.18	0.81 ± 1.34	0.082	3.72 ± 9.24	1.22 ± 1.66	0.263
Blood glucose (mmol/L)	7.83 ± 2.93	7.77 ± 3.24	0.888	8.22 ± 2.68	6.97 ± 1.30	0.067
CRP (mg/L)	16.69 ± 44.41	9.63 ± 14.87	0.181	10.28 ± 22.45	8.50 ± 14.48	0.761
Hemoglobin (g/L)	137.64 ± 19.87	140.13 ± 16.73	0.356	135.07 ± 17.91	139.94 ± 16.69	0.340
Blood platelets (10^9^/L)	240.94 ± 77.47	237.83 ± 56.87	0.759	237.39 ± 94.38	256.78 ± 102.54	0.492
TC (mmol/L)	4.59 ± 1.08	4.48 ± 0.89	0.440	4.67 ± 1.05	4.42 ± 0.82	0.378
TG (mmol/L)	1.50 ± 1.08	3.18 ± 16.50	0.233	1.38 ± 0.99	1.73 ± 2.36	0.448
HDL-C(mmol/L)	1.29 ± 0.38	3.31 ± 16.48	0.150	1.41 ± 0.39	1.48 ± 0.40	0.505
LDL-C (mmol/L)	2.90 ± 1.01	2.68 ± 0.90	0.114	2.87 ± 0.87	2.55 ± 0.79	0.185
HCY (mmol/L)	14.99 ± 9.85	13.84 ± 7.66	0.377	16.07 ± 6.66	15.63 ± 10.73	0.853
Discharge GCS score	11.66 ± 3.62	14.72 ± 0.78	**<0**.**001**	9.67 ± 5.13	14.83 ± 0.38	**<0**.**001**
mRS score after 90 days	4.49 ± 0.64	2.28 ± 0.84	**<0**.**001**	4.78 ± 0.93	2.11 ± 0.90	**<0**.**001**
Maximum cross-sectional diameter of hematoma (mm)	5.24 ± 1.01	4.79 ± 1.01	**0**.**002**	5.65 ± 1.19	4.89 ± 1.05	**0**.**025**
Hematoma volume (ml)	32.18 ± 12.56	23.09 ± 8.87	**<0**.**001**	36.54 ± 12.74	23.88 ± 10.23	**<0**.**001**
Hematoma location			**<0**.**001**			**0**.**001**
Basal ganglia area	112 (0.81)	30 (0.39)		26 (0.72)	5 (0.28)	
Non-basal ganglia area	26 (0.19)	46 (0.61)		10 (0.28)	13 (0.72)	
Hematoma located in the cerebral hemisphere			**<0**.**001**			0.446
Right side	39 (0.28)	52 (0.68)		14 (0.39)	9 (0.50)	
Left side	99 (0.72)	24 (0.32)		22 (0.61)	9 (0.50)	
Whether the hematoma has broken into the ventricles of the brain			0.132			0.084
No	65 (0.47)	44 (0.58)		13 (0.36)	11 (0.61)	
Yes	73 (0.53)	32(0.42)		23(0.64)	7(0.39)	

*p* <0.01 was considered statistically significant.

### 3.2 Screening for clinical and radiomic features

A total of 1,834 radiomic features, including shape features, first order histogram features, and texture features (Figure 2D**)**. There were 360 First Order features, 14 Shape features, 440 GLCM features, 320 GLSZM features, 320 GLRLM features, 100 NGTDM features, and 280 GLDM features. The violin plots showed all features and corresponding *p*-values, and we filtered out features with *p* < 0.05 for the next analysis (Figure 2E). Moreover, we showed the weights of imaging features (Figure 2F).

### 3.3 Performance and validation of clinical and radiomics models

**Figure 3:**
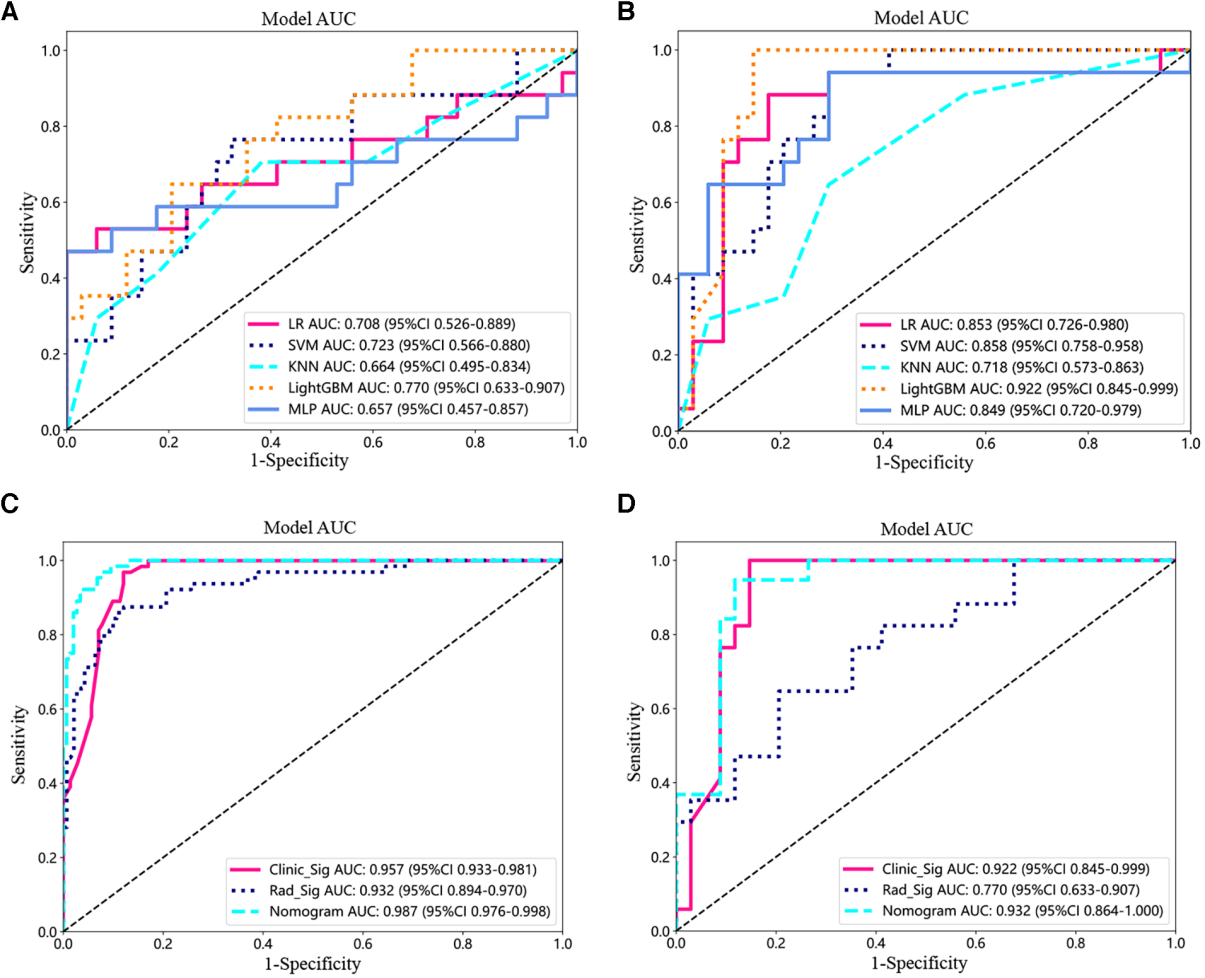
Model testing and validation. (**A,B**) ROC analysis of different machine learning models for imaging features (**A**) and clinical features (**B**) in the test set. (**C,D**) ROC curves for the training (**C**) and testing cohorts **(D**) for the imaging histology model, clinical model, and clinical-imaging histology nomogram.

In the test set, the AUC values of machine learning LR, SVM, KNN, LightGBM, and MLP for radiomics models were 0.708, 0.723, 0.664, 0.770, and 0.657, respectively (Figure 3A). The AUC values of machine learning LR, SVM, KNN, LightGBM, and MLP for the clinical model were 0.853, 0.858, 0.718, 0.922, and 0.849 (Figure 3B).

In the training set, the AUC values for radiomic features modeling using different ML classifiers such as LR, SVM, KNN, LightGBM, and MLP were 0.905, 0.823, 0.851, 0.957 and 0.809, respectively (Table 2). The accuracy, AUC, sensitivity, specificity, positive prediction rate, and negative prediction rate for radiomic features modeling in the training set using the LightGBM algorithm were 0.907, 0.957, 0.969, 0.879, 0.785, and 0.984, respectively (Table 2). The prediction rate and negative prediction rate in the training set using the LightGBM algorithm were 0.907, 0.957, 0.969, 0.879, 0.785, and 0.984, respectively (Table 2). The accuracy, sensitivity, specificity, positive prediction rate, and negative prediction rate of the model for radiomic features in the test set using the LightGBM algorithm were 0.902, 0.922, 1.000, 0.853, 0.773, and 1.000 (Table 2).

**Table 2:** Prediction performance of imaging features by different machine learning algorithms in the training and validation sets.

Machine learning methods	Queue	Accuracy	AUC	95% CI	Sensitivity	Specificity	Positive prediction rate	Negative prediction rate
LR	Training set	0.790	0.905	0.864–0.945	0.938	0.723	0.606	0.962
	Test set	0.843	0.853	0.725–0.980	0.882	0.824	0.714	0.933
SVM	Training set	0.702	0.823	0.766–0.879	0.906	0.610	0.513	0.935
	Test set	0.784	0.858	0.758–0.958	0.941	0.706	0.615	0.960
KNN	Training set	0.761	0.851	0.802–0.900	0.891	0.702	0.576	0.934
	Test set	0.686	0.718	0.572–0.863	0.647	0.750	0.524	0.800
LightGBM	Training set	0.907	0.957	0.932–0.981	0.969	0.879	0.785	0.984
	Test set	0.902	0.922	0.845–0.999	1.000	0.853	0.773	1.000
MLP	Training set	0.717	0.809	0.750–0.868	0.859	0.652	0.529	0.911
	Test set	0.784	0.849	0.720–0.978	0.941	0.706	0.615	0.960

The clinical models were constructed using multiple machine-learning classifiers (Table 3). The AUC values for LR, SVM, KNN, LightGBM, and MLP in the training set were 0.766, 0.863, 0.727, 0.878, and 0.805, respectively. The AUC values in the test set were 0.824, 0.706, 0.647, 0.745, and 0.824, respectively. The accuracy, AUC, sensitivity, specificity, positive prediction rate, and negative prediction rate using the LightGBM model in the training set were 0.878, 0.932, 0.875, 0.879, 0.767, and 0.939, respectively. The accuracy, sensitivity, specificity, positive prediction rate, and negative prediction rate of the modeled radiomic features models in the test set using the LightGBM algorithm were 0.745, 0.770, 0.647, 0.794, 0.611, and 0.818, respectively.

**Table 3:** Prediction performance of imaging features by different machine learning algorithms in the training and validation sets.

Machine learning methods	Queue	Accuracy	AUC	95% CI	Sensitivity	Specificity	Positive prediction rate	Negative prediction rate
LR	Training set	0.766	0.838	0.782–0.893	0.844	0.730	0.587	0.912
	Test set	0.824	0.708	0.525–0.889	0.471	1.000	1.000	0.791
SVM	Training set	0.863	0.929	0.893–0.964	0.906	0.844	0.725	0.952
	Test set	0.706	0.723	0.566–0.880	0.765	0.676	0.542	0.852
KNN	Training set	0.727	0.863	0.812–0.913	0.859	0.667	0.539	0.913
	Test set	0.647	0.664	0.494–0.833	0.706	0.656	0.480	0.808
LightGBM	Training set	0.878	0.932	0.894–0.969	0.875	0.879	0.767	0.939
	Test set	0.745	0.770	0.633–0.906	0.647	0.794	0.611	0.818
MLP	Training set	0.805	0.882	0.832–0.930	0.844	0.787	0.643	0.917
	Test set	0.824	0.657	0.457–0.857	0.471	1.000	1.000	0.791

### 3.4 Predictive model effectiveness evaluation

Next, the prediction effects of the radiomic features model, the clinical model, and the clinical-radiomic nomogram model were compared for patients. In the training set, the AUC of the clinical-radiomic nomogram model was 0.987 (95% CI: 0.976–0.988), which was better than that of the clinical model only 0.957 (0.933–0.981) and the radiomic features model 0.932 (0.894–0.970) (Figure 3C). Also in the test set, the AUC values were 0.932 (95% CI: 0.864–1.000) for the combined nomogram model, 0.922 (95% CI: 0.845–0.999) for the clinical model, and 0.770 (95% CI: 0.633–0.907) for the radiomic features model (Figure 3D).

In the training set, the accuracy, sensitivity, specificity, positive prediction rate, and negative prediction rate of the clinical-radiomic nomogram model were 0.938, 0.969, 0.925, 0.849, and 0.986, respectively (Table 4). In the test set, the accuracy, sensitivity, specificity, positive prediction rate, and negative prediction rate of the clinical-radiomic nomogram model were 0.906, 0.947, 0.882, 0.818, and 0.968, respectively. All of them were better than the separately modeled radiomic features model and clinical model.

**Table 4:** Prediction performance of the three models in the training and validation sets.

Models	Queue	Accuracy	AUC	95% CI	Sensitivity	Specificity	Positive prediction rate	Negative prediction rate
Clinical	Training set	0.907	0.957	0.932–0.981	0.969	0.879	0.785	0.984
	Test set	0.902	0.922	0.845–0.999	1.000	0.853	0.773	1.000
Radiomics	Training set	0.878	0.932	0.894–0.969	0.875	0.879	0.767	0.939
	Test set	0.745	0.770	0.633–0.906	0.647	0.794	0.611	0.818
Nomogram	Training set	0.938	0.987	0.976–0.997	0.969	0.925	0.849	0.986
	Test set	0.906	0.932	0.864–0.999	0.947	0.882	0.818	0.968

In addition, we compared the differences in AUC between the prediction models by Delong's test. In the training set, the combined nomogram model was better than the clinical model and the imaging model in differentiating the prognosis of patients who performed stereotactic hyper encephalic hemorrhage, with a statistically significant difference (*p *= 0.011 and 0.003). In the validation set, the combined nomogram model was superior to the imaging model, with a statistically significant difference (*p *= 0.015).

**Figure 4:**
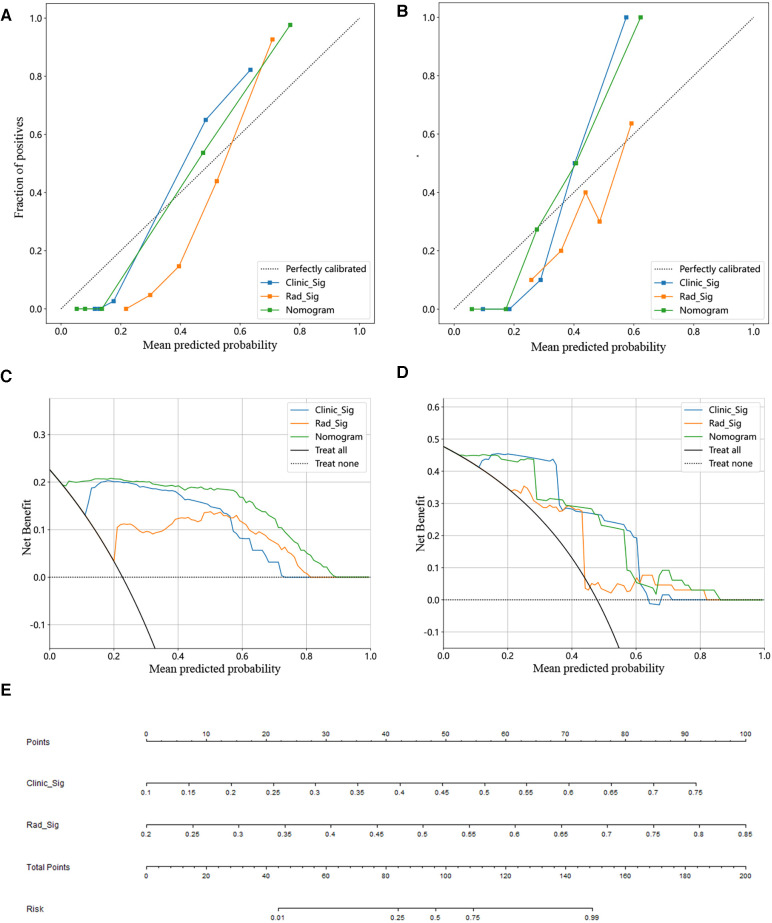
Constructing a clinical-imaging norman model. (**A,B**) Calibration curve for training (**A**) and test set (**B**). (**C,D**) Analysis of decision curves for different models in the training (**C**) and test sets (**D**). (**E**) The nomogram of the clinical features and imaging histological features to build a joint prediction model.

There was good agreement between the training and testing cohorts for the calibration curve prediction and observation line stereotactic prognostic model for patients with hypertensive cerebral hemorrhage, respectively (Figures 4A,B). In this study, we also evaluated each model by DCA, and the decision curve analysis of the combined clinical features, radiomic features, and nomogram model (Figures 4C,D). The preoperative prediction of prognosis in patients with stereotactic hypertensive cerebral hemorrhage applying the combined nomogram model proved to be of better clinical benefit. Finally, a visual Norman diagram model was performed (Figure 4E).

## 4 Discussion

Hypertensive cerebral hemorrhage was a highly prevalent cardio-cerebrovascular disease. It was characterized by acute onset, high risk, high disability, and high morbidity and mortality rates (22). There were more controversies about the surgical treatment modalities for hypertensive cerebral hemorrhage, such as open cranial hematoma removal, small bone window hematoma removal, endoscopic hematoma removal, and CT-guided stereotactic intracranial hematoma removal. In general, the choice of treatment was mainly based on the patient's age, underlying disease, and characteristics of the hemorrhage. However, the principle of treatment was mainly to reduce intracranial pressure, remove the hematoma, and improve patient prognosis (23, 24). Most studies have studied the rebleeding or prognosis of patients with cerebral hemorrhage and have not separately looked at the prognosis of patients who underwent stereotactic hypertensive cerebral hemorrhage.

Minimally invasive borehole drainage was often used in clinical treatment, and the hematoma can be removed without craniotomy, which can reduce the trauma suffered by the body and facilitate the postoperative recovery of the patient. However, there are still some patients with poor prognosis after surgery. Therefore, it is crucial to explore the factors influencing the poor prognosis of patients with hypertensive cerebral hemorrhage after minimally invasive borehole drainage and to intervene effectively in a timely manner to improve the prognosis of patients with hypertensive cerebral hemorrhage (12). In this study, GCS score at admission, maximum transverse diameter of hematoma, hematoma volume, and hematoma location were considered important risk predictors of poor prognosis at 90 days. The GCS scale is a common tool for early neurological examination and its score is positively correlated with neurological function. A study confirmed that early clinically important neurological changes were associated with long-term prognosis in patients with ICH, which is consistent with our findings (25). In clinical studies, it has been shown that early surgical treatment would be an effective treatment for hemorrhagic stroke in some cases (26). However, the surgical criteria and the time of surgical intervention strictly adhered to are usually determined by the GCS score and the change in the amount of intracranial bleeding in ICH (27). Similarly, several studies have demonstrated that preoperative hematoma volume is an influential factor in poor prognosis after borehole drainage in patients with hypertensive cerebral hemorrhage (28).

We built a clinical model to identify patients with line stereotactic HICH with a poor prognosis at 90 days based on these predictors, and the model built on clinical factors achieved suboptimal identification performance with an AUC of 0.957 in the training set and 0.922 in the test set. Although this result suggests that clinical models can also be used to identify patients with HCIH with a poor prognosis, clinical patients with HICH in which predicting poor prognosis based on information about clinical factors alone is not sufficient, Jawed et al. showed that an imaging feature model based on machine learning algorithms constructed for non-enhanced CT scans could analyze the prognosis of ICH patients (29). Meanwhile there was a study by Chao Ma et al. that effectively predicted hematoma expansion by imaging histological features in patients with hypertensive intra-parenchymal hematoma, while imaging histological score provided rapid and quantitative risk assessment for ICH patients with a high discrimination ability to discriminate between hematoma expansion and non-expansion in the training data set with an AUC of 0.89 (95% CI: 0.82–0.96) and an accuracy of 0.85 (30). Studies have shown that imaging features can also predict the prognosis of HICH patients (31). A study by Zhou et al. developed and validated a clinical nomogram based on CT radiomic features for predicting the short-term prognosis of deep cerebral hemorrhage with AUC values of 0.80, 0.79, and 0.70 in the training set, test set, and validation set, respectively (31). Therefore, CT imaging features of HICH are also an important factor involved in the assessment of the functional prognosis of patients (32–34).

Traditional imaging signatures predict the prognostic function of HICH by relying on features such as irregular morphology and inhomogeneous density of the hematoma (black hole sign, swirl sign), but this qualitative feature was usually subjective to human judgment (35, 36). Radiomic features analysis required only a relatively simple delineation of the region of interest and computer-assisted techniques to achieve the assessment of hematoma heterogeneity, thus reducing the physician's empirical requirements for the identification of imaging signs. After radiomic features analysis, we selected 23 of 1,834 imaging radiomic features after screening for association with the 90-day functional outcome of HICH. These 23 radiomic features were derived from shape features, first-order histogram features, and second-order histogram or texture features such as GLCM, GLRLM, GLSZM, and NGTDM describing the heterogeneity, shape, and volume of the hematoma. Changes in hematoma shape and density may indicate multifocal hemorrhage and ongoing active bleeding in different stages (37). Previous studies have identified hematoma volume, shape, and heterogeneity as risk factors associated with the prognosis of patients with ICH (38–40). Various machine learning classifiers were also constructed in order to improve the prediction of the prognosis of patients undergoing stereotactic HICH. Our results show that non-linear machine learning models based on tree models such as LightGBM perform better compared to linear models. It is demonstrated that traditional modeling approaches use linear machine learning models such as LR and SVM, while in the real world, these features often have high order nonlinearity. In addition, radiomic features models can be used to quantitatively reflect the characteristics of the hematoma itself to assess the risk of poor prognosis in patients with ICH. Imagingomics assessment of HICH patients has been shown to have good performance. The machine learning model built with radiomic features had an AUC of 0.932 in the training cohort and 0.770 in the test cohort. also, by further model comparison analysis, the joint model by nomogram had better discriminative performance than the radiomic features model (*p* < 0.05, DeLong test). The AUC of the combined nomogram model was 0.987 in the training set and 0.932 in the test set, demonstrating that the multimodal data construction of radiomic features combined with clinical features was superior to the single-modality model for determining the prognosis of stereotactic cerebral hemorrhage hypertensive patients.

We also performed a comparative analysis of the efficacy of the different models. In the training cohort, this nomogram was significantly better than the clinical model in identifying the 90-day poor functional prognosis of patients with row stereotactic HICH (*p *= 0.011, DeLong test). In addition, the combined nomogram model achieved good performance in identifying patient prognosis in both the training set (AUC = 0.987) and the test set (AUC = 0.932). This result suggests that the use of clinical variables combined with imaging feature composition may become a promising approach to help improve precision medicine. In addition, the combined nomogram model can visually estimate the 90-day functional prognosis of stereotactic HICH patients and individualize the prognosis of patients for risk assessment. Finally, the calibration curves from the training and test cohorts show good agreement between the different models for the training and test cohorts. Also, the nomogram shows good agreement with the actual clinical outcome and the estimated 90-day functional outcome. In addition, this nomogram yields more net gain in predicting 90-day functional outcomes over almost most of the threshold probability range compared to the clinical model, and it can be used as a potentially reliable and reproducible tool to guide clinical practice.

## 5 Conclusion

In this retrospective study, three models were constructed to predict the prognosis of patients with HIHC with stereotactic cerebral hemorrhage, including a radiomic features model, a clinical model, and a combined nomogram model, and the performance of each model was investigated and compared to identify patients with good prognostic function with stereotactic cerebral hemorrhage at 90 days. Our study found that the combined clinical and imaging models had the best diagnostic performance for assessing the prognosis of patients admitted with stereotactic cerebral hemorrhage without relying on extensive experience in identifying imaging signs, making them suitable for use by inexperienced first-line clinicians.

## Data Availability

The raw data supporting the conclusions of this article will be made available by the authors, without undue reservation.
