# Supplementary material for: Predicting the 90-day prognosis of stereotactic brain hemorrhage patients by multiple machine learning using radiomic features combined with clinical features
Source: Front Surg. 2024 Feb 8;11:1344263. doi: 10.3389/fsurg.2024.1344263 (PMC10882084; doi:10.3389/fsurg.2024.1344263)
Supplement: Supplementary file 1 [file Table1.docx]

Supplement Table S1. Modified Rankin Scale (mRS) scale

| Patient Profile | score |
| --- | --- |
| Completely asymptomatic | 0 |
| Not visibly disabled despite symptoms, able to perform all regularly performed duties and activities | 1 |
| Mildly disabled, unable to perform all activities previously performed, but able to manage personal affairs without assistance | 2 |
| Moderate disability, needs some assistance, but no assistance with mobility | 3 |
| Severe disabilities, inability to walk without the assistance of others, and inability to care for their own physical needs | 4 |
| Severely disabled, bedridden, incontinent, requiring constant care and attention | 5 |
| Death | 6 |
