# Supplementary material for: Predicting the 90-day prognosis of stereotactic brain hemorrhage patients by multiple machine learning using radiomic features combined with clinical features
Source: Front Surg. 2024 Feb 8;11:1344263. doi: 10.3389/fsurg.2024.1344263 (PMC10882084; doi:10.3389/fsurg.2024.1344263)
Supplement: Supplementary file 2 [file Table2.docx]

Supplement Table S2. Screening results of imaging features

| Variables | Radiomic features |
| --- | --- |
| A | exponential_glszm_SizeZoneNonUniformityNormalized |
| B | exponential_glszm_ZoneVariance |
| C | gradient_glcm_Idmn |
| D | gradient_glcm_Imc1 |
| E | gradient_glszm_GrayLevelNonUniformity |
| F | lbp_3D_m1_firstorder_Range |
| G | lbp_3D_m1_gldm_SmallDependenceHighGrayLevelEmphasis |
| H | lbp_3D_m1_glszm_GrayLevelVariance |
| I | lbp_3D_m2_firstorder_Range |
| J | lbp_3D_m2_gldm_SmallDependenceHighGrayLevelEmphasis |
| K | lbp_3D_m2_glrlm_ShortRunHighGrayLevelEmphasis |
| L | log_sigma_3_0_mm_3D_glszm_SmallAreaEmphasis |
| M | original_shape_Flatness |
| N | squareroot_glcm_Correlation |
| O | wavelet_HHL_gldm_SmallDependenceLowGrayLevelEmphasis |
| P | wavelet_LHH_glszm_SizeZoneNonUniformityNormalized |
| Q | wavelet_LHL_firstorder_Skewness |
| R | wavelet_LHL_glcm_Correlation |
| S | wavelet_LLL_glrlm_RunLengthNonUniformity |
